# Surgical Correction of Keratinized Mucosa Deficiency Around Dental Implants: A Clinical, Histological and Immunohistochemical Study

**DOI:** 10.3390/dj14050256

**Published:** 2026-04-28

**Authors:** Emil K. Khabirov, Gulshat T. Saleeva, Dmitry E. Tsyplakov, Rinat A. Saleev, Laysan R. Shakirova

**Affiliations:** 1 Department of Prosthetic Dentistry, Kazan State Medical University, Kazan 420012, Russia; 2 General Pathology Department, Kazan State Medical University, Kazan 420012, Russia

**Keywords:** peri-implant soft tissue, keratinized mucosa, dental implants, immunohistochemistry, matrix metalloproteinase-9, TIMP-2, soft tissue augmentation, peri-implant inflammation, extracellular matrix remodeling

## Abstract

**Background/Objectives**: Peri-implant inflammatory complications remain a major cause of late implant failure and are closely associated with the condition of peri-implant soft tissues. Insufficient keratinized attached mucosa has been identified as a potential risk factor for peri-implant inflammation; however, morphological and immunohistochemical validation of soft tissue remodeling following corrective interventions remains limited. The aim of this study was to perform a morphological and immunohistochemical evaluation of a reproducible surgical approach for increasing keratinized attached mucosa around dental implants. **Methods**: A comparative clinical–morphological study included 25 patients undergoing implant-supported prosthetic treatment. Patients were divided into a control group (standard prosthetic protocol without soft tissue augmentation, *n* = 10) and a study group (soft tissue correction using a previously developed technique, *n* = 15). Punch biopsies of peri-implant mucosa were obtained at baseline and prior to definitive prosthetic restoration. Histological examination and immunohistochemical analysis were performed using the semi-quantitative Astaldi–Verga method. Expression of inflammatory markers (MPO, CD3, CD20, CD68), vascular marker CD31, and remodeling markers MMP-9 and TIMP-2 was evaluated. Data were analyzed using the Mann–Whitney U test (*p* < 0.05). **Results**: The study group demonstrated significantly lower expression of inflammatory markers, including MPO, CD68, CD3, and CD20 (*p* < 0.001), and reduced MMP-9 expression (*p* = 0.001) compared with controls. The MMP-9/TIMP-2 balance was more favorable in the study group, suggesting more regulated extracellular matrix remodeling. Histologically, the control group exhibited epithelial disruption and microcirculatory alterations, whereas the study group showed preserved epithelial architecture and reduced inflammatory infiltration. **Conclusions**: Morphological and immunohistochemical assessment suggests that soft tissue correction of keratinized mucosa deficiency may be associated with more favorable early peri-implant soft tissue characteristics, including reduced inflammatory activity and modulation of matrix remodeling. Immunohistochemical markers such as MMP-9 and TIMP-2 may provide additional insight into early soft tissue integration around dental implants. However, these findings should be interpreted with caution due to the exploratory design and short follow-up period.

## 1. Introduction

Peri-implant inflammatory diseases remain one of the leading causes of late implant failure despite the overall high survival rates of dental implants. Chronic inflammation of the peri-implant soft and hard tissues may progress with limited clinical symptoms and is often detected only during follow-up examinations [1,2]. Consequently, identifying biological and local factors that influence peri-implant tissue stability is of considerable clinical relevance.

Among the proposed risk factors, the presence and width of keratinized peri-implant mucosa have attracted particular attention [3]. Several clinical studies suggest that insufficient keratinized mucosa is associated with impaired plaque control, increased mucosal mobility, soft tissue discomfort, and higher susceptibility to inflammatory changes [4,5,6,7]. Moreover, peri-implant soft tissue conditions are closely linked to marginal bone stability and long-term peri-implant health [2]. However, the necessity of keratinized mucosa for maintaining peri-implant health remains debated, as some reports indicate that acceptable clinical outcomes may still be achieved in its absence under strict plaque control [8]. The design of the emergence profile of dental implant is also a key determinant of successful esthetic results and the maintenance of peri-implant health and tissue stability [9]. Emergence angles < 30° and a concave/straight profile led to lower peri-implantitis risk [10,11]. This ongoing controversy underscores the need for additional biologically oriented investigations.

Soft tissue augmentation procedures have been widely implemented to increase the width and thickness of keratinized mucosa around implants. Systematic reviews and consensus reports confirm that such interventions may improve soft tissue stability and patient comfort [12,13,14]. Nevertheless, most available studies focus primarily on clinical parameters, while morphological and molecular aspects of soft tissue remodeling remain insufficiently characterized.

Integrating transcriptomic, metagenomic, and bioinformatic biomarkers with machine learning may enable earlier and more accurate diagnosis of peri-implantitis [15]. Matrix metalloproteinases (MMPs), particularly MMP-9, play a central role in extracellular matrix degradation, inflammatory cell migration, and angiogenesis, whereas tissue inhibitors of metalloproteinases (TIMPs) regulate their proteolytic activity [16,17]. An imbalance between MMPs and TIMPs has been implicated in periodontal and peri-implant tissue destruction [18,19]. Despite growing interest in biochemical markers of inflammation, immunohistochemical validation of peri-implant soft tissue remodeling following corrective interventions is still limited.

Therefore, the aim of the present study was to perform a morphological and immunohistochemical evaluation of a reproducible surgical approach for the correction of keratinized attached mucosa deficiency around dental implants. By analyzing inflammatory cell infiltration, vascular alterations, and the balance between MMP-9 and TIMP-2 expression, this study seeks to provide objective biological evidence regarding soft tissue integration and peri-implant stability.

## 2. Materials and Methods

### 2.1. Study Design and Ethical Approval

A comparative clinical–morphological study was conducted to evaluate peri-implant soft tissue remodeling during the prosthetic stage of implant treatment using two different clinical protocols. The study was conducted in accordance with the Declaration of Helsinki and approved by the Local Ethics Committee of Kazan State Medical University (Protocol No. 4/2024, approved on 23 April 2024). Written informed consent was obtained from all participants prior to inclusion in the study.

### 2.2. Study Population

Twenty-five patients (aged 30–64 years) undergoing implant-supported prosthetic rehabilitation were included. Inclusion criteria comprised: (1) width of keratinized attached mucosa < 3 mm at the time of prosthetic stage initiation; (2) continuous dentition (excluding third molars and implant sites); and (3) absence of systemic diseases affecting wound healing. Exclusion criteria included severe alveolar ridge atrophy, ongoing orthodontic treatment, significant occlusal abnormalities, and smoking.

Patients were allocated into two groups based on clinical indications and treatment planning without randomization. This non-randomized allocation may introduce selection bias, which is acknowledged as a limitation of the study.

Control Group (*n* = 10): Standard prosthetic protocol without soft tissue augmentation.

Study Group (*n* = 15): Soft tissue correction performed according to a proposed technique for increasing keratinized attached mucosa at the stage of temporary prosthetic restoration.

### 2.3. Clinical Procedure

In the control group, prosthetic treatment was performed according to a conventional protocol following healing abutment placement. Impressions were obtained two weeks after abutment placement, followed by fabrication and placement of definitive zirconia-based restorations.

**Figure 1:**
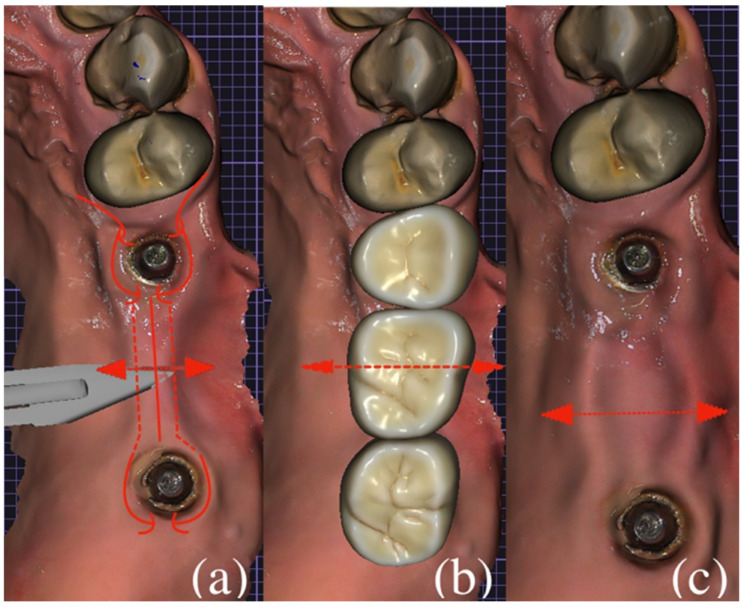
Schematic representation of the surgical protocol. (**a**) Design of converging vertical and semilunar incisions within the zone of keratinized attached mucosa around dental implants. Arrows indicate the direction of flap mobilization. (**b**) Placement of the provisional prosthetic construction with concave subgingival contour and ovate pontic design providing three-dimensional soft tissue support. (**c**) Final position of the displaced gingival tissues demonstrating expansion of the keratinized mucosa zone.

In the study group, soft tissue correction was performed at the stage of temporary prosthetic restoration using a reproducible surgical approach aimed at increasing keratinized mucosa width. The surgical workflow is schematically illustrated in Figure 1. At the first prosthetic appointment, following removal of the standard healing abutments, an intraoral optical impression was obtained with registration of the implant emergence profiles. Based on the digital workflow, full-contour provisional fixed dental prostheses were fabricated using three-dimensional printing technology. The subgingival portion of the provisional restorations in the implant area was designed with a concave (cup-shaped) configuration, while the pontic region was designed with an ovate form to allow soft tissue adaptation.

**Figure 2:**
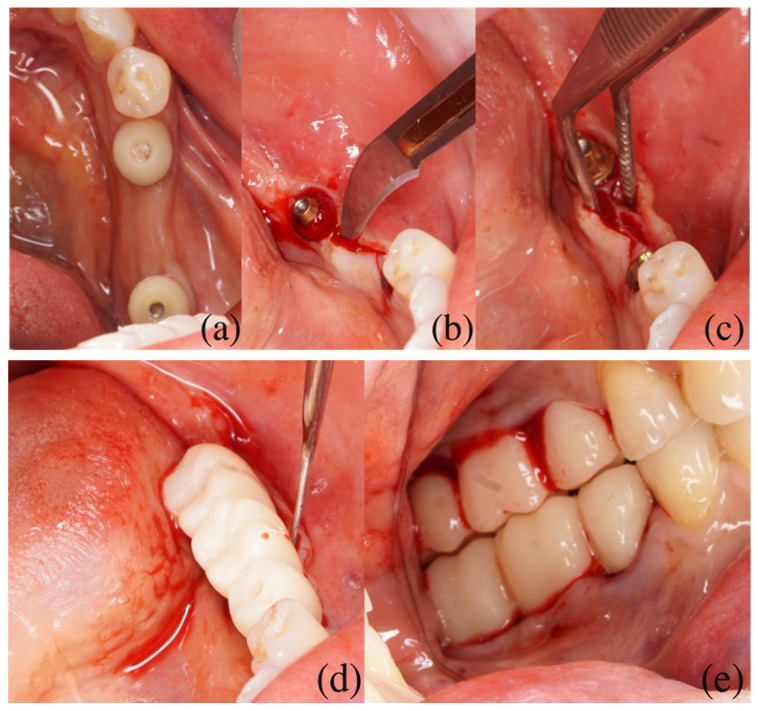
Clinical sequence of the proposed technique. (**a**) Baseline clinical situation after osseointegration of dental implants with individualized healing abutments shaping the peri-implant soft tissues. (**b**) Two converging vertical incisions extending to the periosteum from the mesial and distal aspects of the implant site. (**c**) Split-thickness flap mobilization with vestibular and oral displacement of the gingival tissues. (**d**) Adaptation and apical displacement of mobilized flaps around the provisional prosthesis with concave subgingival contour. (**e**) Final intraoperative view demonstrating vestibular positioning of the gingival tissues around the provisional restoration.

At the second appointment, prior to placement of the provisional constructions, surgical correction of keratinized attached mucosa deficiency was performed under infiltration anesthesia. After removal of the individualized healing abutments, two converging vertical incisions were made extending from the adjacent mesial and distal teeth toward the implant site, reaching the periosteum while preserving the interdental papillae. The incisions were performed to the level of the periosteum to allow controlled tissue displacement while minimizing flap mobility. The incisions were continued along the implant emergence profile. At a distance of 1–3 mm from the implant shaft, both vestibularly and orally, two semilunar incisions were performed parallel to the alveolar bone to a depth of approximately 3–4 mm, creating a split-thickness flap. The incision was then extended along the crest of the alveolar ridge on both vestibular and lingual/palatal aspects in the projection of the future pontic region up to the borders of the adjacent implant site. The surgical approach was designed to achieve limited flap mobilization without extensive detachment, preserving the intrinsic vascular supply and structural stability of the tissues. No sutures were applied, as stabilization of the displaced soft tissues was achieved by the contour of the provisional prosthesis. Clinical sequence of the proposed technique is shown in Figure 2.

The mobilized gingival tissue was gently displaced vestibularly and orally and adapted around the pre-fabricated provisional restoration. The concave subgingival contour in the implant region and the ovate pontic design provided three-dimensional soft tissue support and stabilization without additional grafting materials.

Within 10–14 days, the peri-implant mucosa adapted to the prosthetic contours, with formation of the emergence profile and clinical enlargement of the keratinized attached mucosa zone with pale-pink appearance.

### 2.4. Biopsy Procedure

Punch biopsies (Ø = 1 mm) of peri-implant mucosa were obtained twice in both groups:

(1) At baseline prior to intervention;
(2) Before placement of the definitive prosthesis (14 days after initiation of treatment).

Biopsy samples were collected from the region corresponding to the implant emergence profile or the future pontic site. The 14-day observation period was selected to assess early soft tissue healing and initial remodeling processes following intervention.

### 2.5. Histological Processing

Histological and immunohistochemical evaluations were performed by a blinded examiner who was not aware of group allocation. Specimens were fixed in 10% neutral buffered formalin, dehydrated in graded ethanol solutions, cleared in xylene, and embedded in paraffin. Sections (4–5 μm) were prepared using a Leica SM 2000R microtome (Leica Microsystems, Wetzlar, Germany) and stained with hematoxylin–eosin and Van Gieson stain for morphological assessment.

### 2.6. Immunohistochemical Analysis

Immunohistochemical staining was performed using the LSAB+ System-HRP method with diaminobenzidine as chromogen. The primary antibodies used in this study are summarized in Table 1.

Immunohistochemical results were evaluated using the semi-quantitative Astaldi–Verga method [20]. This method assesses enzyme activity or immunohistochemical reactions based on both the number of positively stained cells and staining intensity. In each specimen, 100 cells were examined and classified according to staining intensity as follows: ++++ (very strong), +++ (strong), ++ (moderate), + (weak), and - (no staining).

The reaction index for each case was calculated using the formula:

(4a + 3b + 2c + 1d + 0e)/100

where a–e represent the number of cells in each staining category.

### 2.7. Statistical Analysis

Due to the exploratory design of this clinical–morphological study, a formal sample size calculation was not performed. The normality of distribution for continuous variables was assessed using the Shapiro–Wilk test. A statistically significant Shapiro–Wilk test result (*p* ≤ 0.05) indicated deviation from normal distribution. As most variables demonstrated non-normal distribution, descriptive statistics were presented as median (Me) with lower (Q1) and upper (Q3) quartiles in the format Me [Q1–Q3]. Intergroup comparisons between independent samples were performed using the nonparametric Mann–Whitney U test. The level of statistical significance was set at 5% (*p* ≤ 0.05). Statistical analysis was performed using STATISTICA 13.0 software (StatSoft Inc., Tulsa, OK, USA).

## 3. Results

### 3.1. Group I

#### 3.1.1. Clinical and Histological Findings

In general, the histological structure of the peri-implant mucosa approximated normal gingival architecture. A stratified squamous epithelium was identified, which was non-keratinized in the area corresponding to the implant emergence profile. Keratinization was present at the marginal gingival edge, with clearly distinguishable basal, spinous, granular, and keratinized layers.

**Figure 3:**
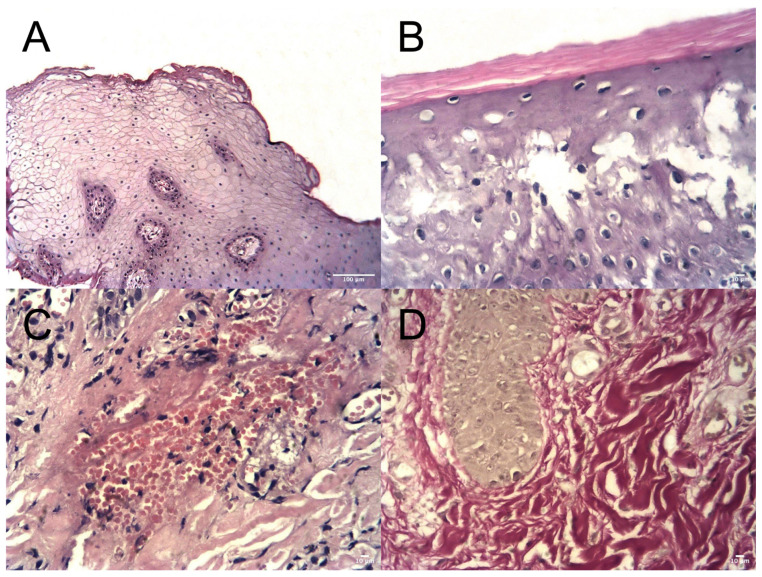
Histological features of peri-implant mucosa in the control group (Group I). (**A**) Thinning and disruption of the keratinized epithelial layer (H&E, ×200, scale bar = 100 µm). (**B**) Focal epithelial disruption (H&E, ×400, scale bar = 10 µm). (**C**) Erythrocyte extravasation beyond the vascular lumen (H&E, ×400, scale bar = 10 µm). (**D**) Sclerosis of the lamina propria with replacement by dense fibrous connective tissue (Van Gieson, ×400, scale bar = 10 µm).

In several specimens, thinning of the keratinized layer with partial disruption of its integrity was observed (Fi). Focal epithelial disruption was also detected (Figure 3B).

The lamina propria consisted of loose fibrous connective tissue forming papillae extending into the epithelium, with deeper areas composed of dense irregular connective tissue. Vascular congestion and dilation of microvessels were observed, in some cases accompanied by erythrocyte extravasation (Figure 3C).

Lymphohistiocytic inflammatory infiltrates were present within the lamina propria. Areas of sclerosis with replacement of loose connective tissue by dense fibrous connective tissue were also identified (Figure 3D).

#### 3.1.2. Immunohistochemical Findings

Immunohistochemistry demonstrated epithelial expression of pan-cytokeratins, although staining was uneven in some specimens. The epithelial basement membrane was delineated by anti-collagen type IV antibodies and was generally continuous, with focal thickening or splitting.

**Figure 4:**
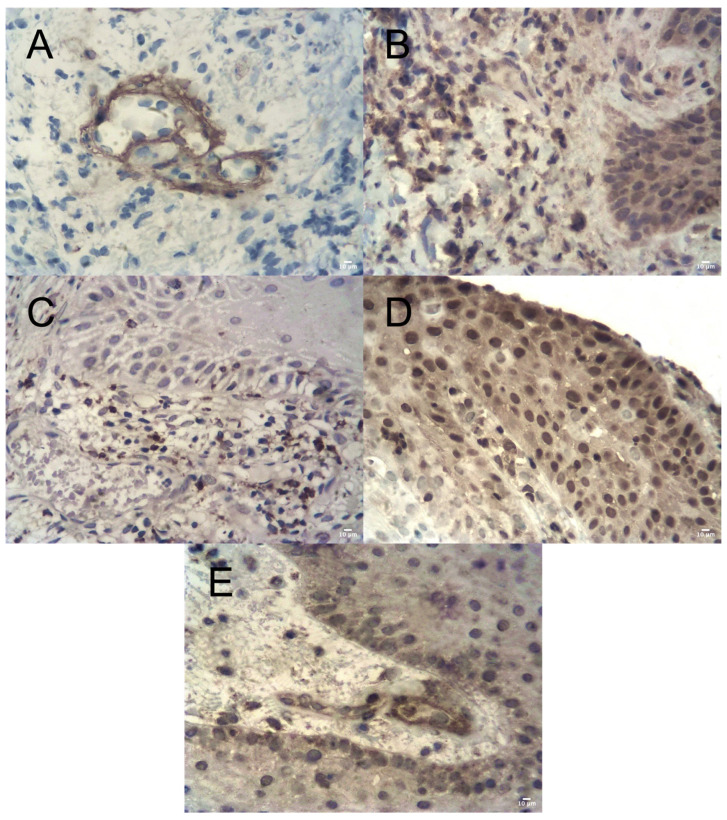
Immunohistochemical features of peri-implant mucosa in the control group (Group I). (**A**) Thickening and splitting of the vascular basement membrane (anti-collagen IV IHC, LSAB, ×400, scale bar = 10 µm). (**B**) Neutrophilic infiltration (anti-MPO IHC, LSAB, ×400, scale bar = 10 µm). (**C**) Macrophage infiltration (anti-CD68 IHC, LSAB, ×400, scale bar = 10 µm). (**D**) High MMP-9 expression (IHC, LSAB, ×400, scale bar = 10 µm). (**E**) TIMP-2 expression (IHC, LSAB, ×400, scale bar = 10 µm).

Within the microcirculatory bed of the lamina propria, CD31-positive endothelial cells and collagen type IV-positive basement membranes were identified. In some vessels, endothelial protrusion into the lumen and splitting with thickening of basement membranes were observed (Figure 4A). Areas of fibrosis demonstrated strong vimentin expression.

The inflammatory infiltrate was predominantly composed of neutrophilic leukocytes and macrophages (Figure 4B,C). T- and B-lymphocytes were present in lower quantities. MMP-9 expression was detected in both the epithelial layer and the underlying connective tissue (Figure 4D). TIMP-2 expression was also identified in these compartments (Figure 4E). The semi-quantitative index indicated a shift in the MMP-9/TIMP-2 ratio toward MMP-9 predominance.

### 3.2. Semi-Quantitative Analysis

The semi-quantitative immunohistochemical indices are presented in Table 2.

Significant differences between Group I and Group II were observed for vimentin (*p* = 0.0096), MPO (*p* < 0.001), CD3 (*p* < 0.001), CD20 (*p* < 0.001), CD68 (*p* < 0.001), and MMP-9 (*p* = 0.0015). No statistically significant differences were detected for pan-cytokeratins (*p* = 0.8489), CD31 (*p* = 0.0545), or TIMP-2 (*p* = 0.1963). Detailed results are provided in Table 2.

**Figure 5:**
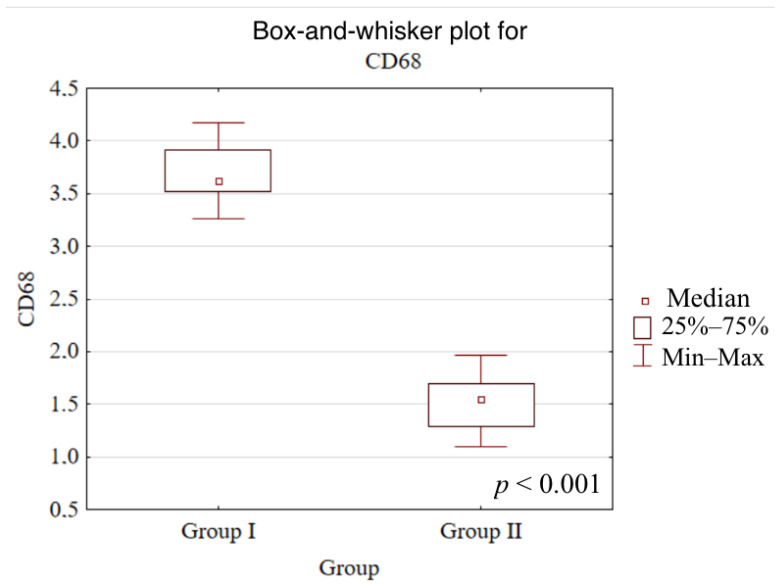
Box-and-whisker plot comparing Groups I and II for CD68 semi-quantitative immunohistochemical index (median, interquartile range, minimum–maximum).

**Figure 6:**
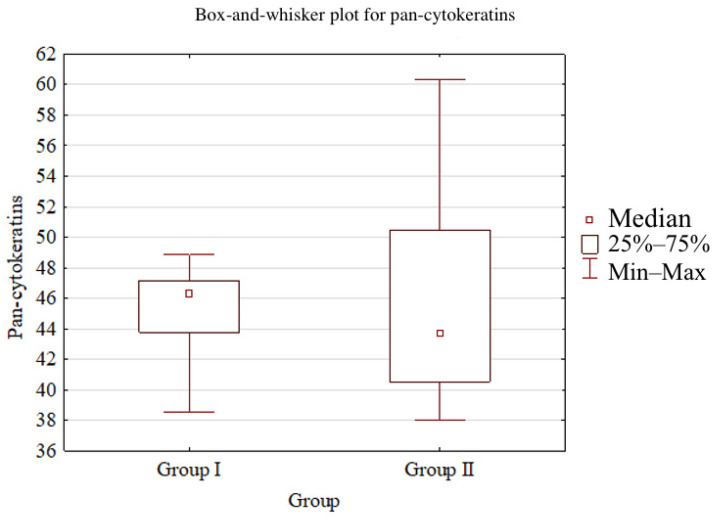
Box-and-whisker plot comparing Groups I and II for pan-cytokeratin semi-quantitative immunohistochemical index (median, interquartile range, minimum–maximum).

Box-and-whisker plots comparing Groups I and II for CD68 and pan-cytokeratins are presented below (Figure 5 and Figure 6).

### 3.3. Group II

#### 3.3.1. Histological Findings

**Figure 7:**
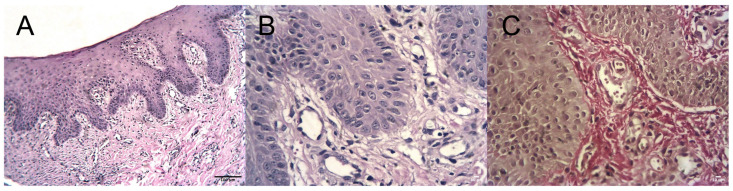
Histological features of peri-implant mucosa in the study group (Group II). (**A**) Normal histological structure of peri-implant mucosa (H&E, ×200, scale bar = 100 µm). (**B**) Higher magnification of Figure 7A (H&E, ×400, scale bar = 10 µm). (**C**) Loose fibrous connective tissue of the papillary lamina propria (Van Gieson, ×400, scale bar = 10 µm).

The structure of the peri-implant mucosa corresponded to normal histological architecture in both the epithelial layer and the lamina propria (Figure 7A,B). All layers of the stratified squamous epithelium were clearly defined. No focal epithelial disruption or keratin layer destruction was observed.

The papillary lamina propria consisted of loose fibrous connective tissue without sclerotic changes (Figure 7C). Dense irregular connective tissue was confined to deeper regions, consistent with normal gingival histology. Microcirculatory alterations were not identified. Lymphohistiocytic infiltrates were present but less pronounced compared with Group I.

#### 3.3.2. Immunohistochemical Findings

**Figure 8:**
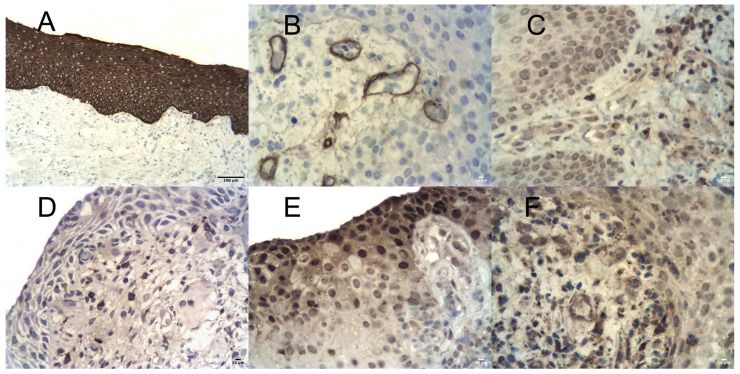
Immunohistochemical features of peri-implant mucosa in the study group (Group II). (**A**) Uniform epithelial pan-cytokeratin expression (IHC, LSAB, ×200, scale bar = 100 µm). (**B**) Normal vascular basement membranes (anti-collagen IV IHC, LSAB, ×400, scale bar = 10 µm). (**C**) Isolated neutrophilic leukocytes (anti-MPO IHC, LSAB, ×400, scale bar = 10 µm). (**D**) Sparse macrophages (anti-CD68 IHC, LSAB, ×400, scale bar = 10 µm). (**E**) MMP-9 expression (IHC, LSAB, ×400, scale bar = 10 µm). (**F**) High TIMP-2 expression (IHC, LSAB, ×400, scale bar = 10 µm).

Uniform epithelial expression of pan-cytokeratins was observed (Figure 8A). The epithelial basement membrane was clearly delineated by collagen type IV staining without structural alterations. CD31 and collagen type IV expression in blood vessels corresponded to normal endothelial and basement membrane morphology (Figure 8B). Within the lamina propria, neutrophilic leukocytes and macrophages were detected, although their numbers were markedly lower compared with Group I (Figure 8C,D).

MMP-9 and TIMP-2 expression was observed in epithelial and connective tissue components (Figure 8E,F). Compared with Group I, MMP-9 expression was lower. The semi-quantitative index demonstrated a shift in the MMP-9/TIMP-2 ratio toward TIMP-2 predominance.

## 4. Discussion

### 4.1. Principal Findings

The present study suggests that morphological and immunohistochemical assessment provides additional insight into peri-implant soft tissue remodeling at the prosthetic stage beyond clinical evaluation alone. The proposed method for increasing keratinized attached mucosa was associated with preservation of epithelial integrity, reduced inflammatory cell infiltration, and a more balanced expression of matrix remodeling markers compared with the conventional protocol. These findings should be interpreted with caution due to the exploratory design and short observation period.

These findings are consistent with the concept that peri-implant soft tissue stability is closely related to both structural integrity and inflammatory control, which are key determinants of long-term peri-implant health [1,2].

### 4.2. Inflammation and Microcirculatory Alterations

In the control group, epithelial disruption, vascular congestion, erythrocyte extravasation, and lymphohistiocytic infiltration were observed. These morphological changes may represent a tissue environment characterized by sustained inflammatory activation.

Previous consensus reports and clinical studies have emphasized the importance of peri-implant soft tissue health in preventing inflammatory complications and maintaining marginal bone stability [1,2]. Increased vascular density and microvascular alterations have been described in inflamed gingival tissues, reflecting active angiogenesis and inflammatory progression [21]. The microcirculatory alterations observed in the control group are consistent with these observations and may indicate ongoing inflammatory remodeling.

In contrast, the study group demonstrated preserved epithelial architecture and absence of pronounced vascular disturbances, suggesting a more stable peri-implant mucosal barrier.

### 4.3. MMP-9/TIMP-2 Balance and Connective Tissue Remodeling

Particular attention was given to the expression of MMP-9 and its inhibitor TIMP-2. Matrix metalloproteinases play a central role in extracellular matrix degradation, inflammatory cell migration, and angiogenesis [16,19,22]. Elevated MMP activity has been associated with periodontal and peri-implant tissue breakdown.

In the present study, higher MMP-9 expression and a shift in the MMP-9/TIMP-2 ratio toward proteolytic predominance were observed in the control group. This pattern is consistent with an environment characterized by enhanced matrix turnover and potential connective tissue destabilization. In contrast, the study group exhibited lower MMP-9 expression and a relative shift toward TIMP-2, suggesting a more regulated remodeling process.

The biological relevance of these findings is supported by previous reports indicating that neutrophils represent a major source of MMP-9 and that activated macrophages contribute to metalloproteinase upregulation [17,23]. The higher MPO-positive neutrophil and CD68-positive macrophage indices in the control group may therefore explain the increased MMP-9 expression detected in this group. Additionally, elevated levels of neutrophil extracellular traps have been implicated in epithelial barrier dysfunction and altered keratinization in periodontal inflammation [24]. These mechanisms may partially account for the epithelial alterations observed in the control group.

### 4.4. Clinical Implications

Soft tissue management around dental implants has been recognized as a critical factor influencing peri-implant health and patient comfort [7,8]. Several conventional soft tissue augmentation techniques, such as free gingival grafts, connective tissue grafts, and collagen matrix substitutes, have demonstrated clinical efficacy in increasing the width of keratinized mucosa [12,13,25]. However, these approaches are often associated with additional surgical morbidity, donor site complications, or material-related limitations.

In contrast, the present technique is performed at the prosthetic stage without the use of grafting materials and relies on controlled soft tissue displacement combined with prosthetic contouring. This approach may represent a minimally invasive alternative for managing keratinized mucosa deficiency.

At the same time, most existing studies primarily focus on clinical outcomes, while histological and immunohistochemical validation of soft tissue quality remains limited. Therefore, the present findings may add a biological perspective to the evaluation of peri-implant soft tissue augmentation. Nevertheless, direct comparative clinical studies with established augmentation techniques are required to confirm the clinical effectiveness and long-term benefits of the proposed approach.

### 4.5. Limitations and Future Research

This study has several limitations. The sample size was relatively small and no formal sample size calculation was performed due to the exploratory nature of the study. Second, the non-randomized allocation of patients may introduce selection bias. Third, follow-up was limited to 14 days and reflects only early stages of soft tissue healing. Fourth, clinical parameters such as probing depth and bleeding on probing were not assessed, limiting the clinical interpretation of the findings. Furthermore, immunohistochemical assessment was performed using a semi-quantitative visual scoring system, which may introduce observer-dependent variability.

Future research should include larger patient cohorts, longer observation periods, and quantitative digital image analysis to improve precision in assessing marker expression. Longitudinal studies correlating immunohistochemical findings with long-term clinical outcomes, including peri-implantitis incidence and marginal bone changes, would further clarify the clinical relevance of the observed remodeling patterns.

## 5. Conclusions

The present study suggests that morphological and immunohistochemical evaluation provides an objective characterization of peri-implant soft tissue remodeling at the prosthetic stage of treatment. Semi-quantitative assessment of inflammatory and extracellular matrix remodeling markers, including MMP-9 and TIMP-2, revealed significant intergroup differences in tissue response associated with different clinical protocols for managing keratinized attached mucosa.

The proposed method for correcting keratinized mucosa deficiency around dental implants was associated with preservation of epithelial integrity, reduced inflammatory infiltrate, and a more balanced MMP-9/TIMP-2 expression profile. These findings may indicate more controlled connective tissue remodeling and suggest a potential improvement in the biological stability of the peri-implant soft tissue barrier. However, these results should be interpreted with caution due to the exploratory design of the study, the relatively small sample size, and the short follow-up period. Further studies with larger cohorts and long-term clinical evaluation are required to confirm the clinical relevance of these findings.

## 6. Patents

The surgical protocol described in this study is based on a previously developed clinical approach for managing keratinized mucosa deficiency around dental implants.

## Figures and Tables

**Table 1:** Primary antibodies used for immunohistochemical analysis.

Antigen	Clone	Target	Dilution	Manufacturer
Pan-cytokeratins (CK)	AE1/AE3	Epithelial cells	1:300	Lab Vision (Fremont, CA, USA)
Myeloperoxidase (MPO)	Polyclonal (RB-373-A)	Neutrophils	1:800	Dako (Glostrup, Denmark)
Vimentin	V9	Fibroblasts	1:300	Lab Vision (Fremont, CA, USA)
CD3	SP7	T lymphocytes	1:150	Lab Vision (Fremont, CA, USA)
CD20	L26	B lymphocytes	1:250	Lab Vision (Fremont, CA, USA)
CD68	PGM1	Macrophages	1:200	BioGenex (Fremont, CA, USA)
CD31	9611	Vascular endothelium	1:20	BioGenex (Fremont, CA, USA)
Collagen IV	PHM-12 + CIV22	Basement membranes	1:150	Lab Vision (Fremont, CA, USA)
MMP-9	PR066	Connective tissue cells expressing matrix metalloproteinase-9	1:50	Diagnostic Biosystems (Pleasanton, CA, USA)
TIMP-2	3A4	Connective tissue cells expressing tissue inhibitor of metalloproteinases-2	1:75	Diagnostic Biosystems (Pleasanton, CA, USA)

**Table 2:** Semi-quantitative immunohistochemical indices in peri-implant mucosa in Groups I and II (Me [Q1–Q3]). Intergroup comparisons were performed using the Mann–Whitney U test.

Marker	Group I Me [Q1–Q3]	Group II Me [Q1–Q3]	*p*-Value
Pan-cytokeratins	46.3 [43.8–47.2]	43.7 [40.5–50.5]	0.8489
Vimentin	39.1 [34.8–41.0]	32.5 [31.6–37.8]	0.0096
MPO	3.6 [3.5–4.0]	1.1 [0.9–1.2]	<0.001
CD3	1.2 [1.0–1.2]	0.5 [0.5–0.5]	<0.001
CD20	2.1 [1.9–2.3]	0.6 [0.5–0.7]	<0.001
CD68	3.6 [3.5–3.9]	1.6 [1.3–1.7]	<0.001
CD31	7.7 [6.7–10.2]	6.4 [4.5–8.4]	0.0545
MMP-9	56.6 [51.3–62.9]	44.9 [38.7–46.9]	0.0015
TIMP-2	31.6 [27.8–32.5]	28.2 [27.1–32.2]	0.1963

## Data Availability

The data presented in this study are available from the corresponding author upon reasonable request. The data are not publicly available due to privacy and ethical restrictions.
